# Thrombin induces Egr-1 expression in fibroblasts involving elevation of the intracellular Ca^2+ ^concentration, phosphorylation of ERK and activation of ternary complex factor

**DOI:** 10.1186/1471-2199-10-40

**Published:** 2009-05-11

**Authors:** Oliver G Rössler, Gerald Thiel

**Affiliations:** 1 Department of Medical Biochemistry and Molecular Biology, University of Saarland Medical Center, D-66421 Homburg, Germany

## Abstract

**Background:**

The serine protease thrombin catalyzes fibrin clot formation by converting fibrinogen into fibrin. Additionally, thrombin stimulation leads to an activation of stimulus-responsive transcription factors in different cell types, indicating that the gene expression pattern is changed in thrombin-stimulated cells. The objective of this study was to analyze the signaling cascade leading to the expression of the zinc finger transcription factor Egr-1 in thrombin-stimulated lung fibroblasts.

**Results:**

Stimulation of 39M1-81 fibroblasts with thrombin induced a robust and transient biosynthesis of Egr-1. Reporter gene analysis revealed that the newly synthesized Egr-1 was biologically active. The signaling cascade connecting thrombin stimulation with Egr-1 gene expression required elevated levels of cytosolic Ca^2+^, the activation of diacylgycerol-dependent protein kinase C isoenzymes, and the activation of extracellular signal-regulated protein kinase (ERK). Stimulation of the cells with thrombin triggered the phosphorylation of the transcription factor Elk-1. Expression of a dominant-negative mutant of Elk-1 completely prevented Egr-1 expression in stimulated 39M1-81 cells, indicating that Elk-1 or related ternary complex factors connect the intracellular signaling cascade elicited by activation of protease-activated receptors with transcription of the Egr-1 gene. Lentiviral-mediated expression of MAP kinase phosphatase-1, a dual-specific phosphatase that dephosphorylates and inactivates ERK in the nucleus, prevented Elk-1 phosphorylation and Egr-1 biosynthesis in thrombin stimulated 39M1-81 cells, confirming the importance of nuclear ERK and Elk-1 for the upregulation of Egr-1 expression in thrombin-stimulated lung fibroblasts. 39M1-81 cells additionally express M_1 _muscarinic acetylcholine receptors. A comparison between the signaling cascades induced by thrombin or carbachol showed no differences, except that signal transduction via M_1 _muscarinic acetylcholine receptors required the transactivation of the EGF receptor, while thrombin signaling did not.

**Conclusion:**

This study shows that stimulus-transcription coupling in thrombin-treated lung fibroblasts relies on the elevation of the intracellular Ca^2+^-concentration and the activation of PKC and ERK. In the nucleus, ternary complex factors function as key proteins linking the intracellular signaling cascade with enhanced transcription of the Egr-1 gene. This study further shows that the dominant-negative Elk-1 mutant is a valuable tool to study Elk-1-mediated gene transcription.

## Background

The serine protease thrombin (EC 3.4.21.5) is a key enzyme in tissue haemostasis. Thrombin catalyzes fibrin clot formation by converting fibrinogen into fibrin. Moreover, platelets are stimulated by thrombin to aggregation and secretion. In addition, thrombin functions as a mitogen for several cell types, including smooth muscle cells, fibroblasts, and keratinocytes [1-4]. Thrombin binds to seven-transmembrane-spanning G-protein coupled receptors termed protease-activated receptors (PARs). These receptors are activated via proteolysis. Thrombin cleaved off N-terminal amino acids from the extracellular part of the receptor, generating a new N-terminus for the receptor protein that functions as a "tethered ligand" capable of activating the receptor [5-8].

Thrombin stimulation has been shown to activate stimulus-responsive transcription factors in different cell types, suggesting that activation of protease-activated receptors results in changes in gene transcription. In fact, activation of c-Fos and c-Jun has been reported upon stimulation with thrombin in CCL39 and NIH3T3 fibroblasts, endothelial and astrocytoma cells [2,9-12]. The transcription factor NF-κB is activated in thrombin-stimulated microglia cells [13]. Expression of Egr-1 is also regulated by thrombin in endothelial cells and keratinocytes [4,14]. The activation of Egr-1 is regulated via its biosynthesis, in contrast to other stimulus-induced transcription factors such as NF-κB or CREB that are activated via nuclear translocation or phosphorylation. In unstimulated cells, only low levels of Egr-1 are detectable. Upon stimulation by extracellular signaling molecules including growth factors, hormones, and neurotransmitters, the biosynthesis of Egr-1 is induced [15,16]. Egr-1 couples extracellular signals with long-term responses by altering the gene expression pattern of Egr-1 target genes.

We have analyzed the signaling cascade leading to enhanced Egr-1 biosynthesis in thrombin-stimulated 39M1-81 lung fibroblasts. These cells are derived from CCL39 Chinese hamster lung fibroblasts, a cellular model system that has often been used to elucidate the intracellular signaling cascade following stimulation with thrombin. 39M1-81 cells express – in addition to protease-activated receptors – type M_1 _muscarinic acetylcholine receptors. Thus, we were able to compare two signaling cascades that share the activation of phospholipase C downstream of receptor stimulation [9]. Here, we show that the intracellular signaling cascade connecting protease-activated receptors with Egr-1 gene transcription requires a rise of the intracellular Ca^2+ ^ion concentration, the activation of diacylgycerol-dependent PKC isoenzymes, the activation of ERK and the phosphorylation and activation of the transcription factor Elk-1. In contrast to M_1 _muscarinic acetylcholine receptor activation, no transactivation of the EGF receptor was required to connect thrombin stimulation with enhanced Egr-1 gene transcription.

## Results

### Biosynthesis of Egr-1 in thrombin and carbachol-treated 39M1-81 cells

**Figure 1:**
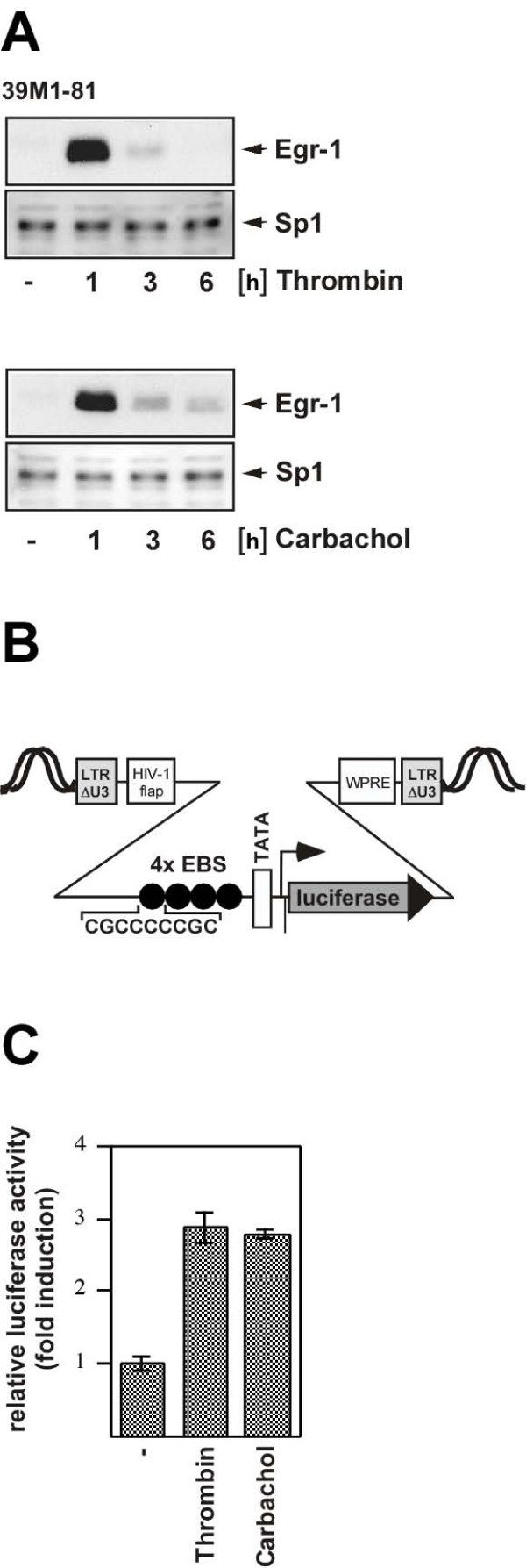
**Stimulation of 39M1-81 fibroblasts with either thrombin or carbachol induces the biosynthesis of biologically active Egr-1**. (A) 39M1-81 cells were serum-starved for twenty-four hours and then treated with thrombin (1 U/ml) or carbachol (100 μM) for 1, 3 or 6 hours as indicated and expression of Egr-1 was analyzed by immunoblotting. As a control, expression of the zinc finger protein Sp1 was analyzed. (B) Schematic representation of integrated provirus encoding an Egr-1 responsive luciferase reporter gene consisting of four binding sites for Egr-1 derived from the human synapsin I promoter, the human immunodeficiency virus TATA box, the adenovirus major late promoter initiator element and the luciferase open reading frame. (C) The newly synthesized Egr-1 protein is biologically active. 39M1-81 cells were infected with a recombinant lentivirus encoding the Egr-1 responsive reporter gene. The infected cells were stimulated with thrombin or carbachol for 16 hours. Cell extracts were prepared and analyzed for luciferase activities which were normalized to the protein concentrations. Each experiment illustrated here and in all subsequent figures was repeated a minimum of three times.

39M1-81 lung fibroblasts were serum-starved for 24 hours and then incubated with either thrombin to stimulate protease-activated receptors or carbachol to stimulate M_1 _muscarinic acetylcholine receptors. The cells were harvested 1, 3, and 6 hours after stimulation, nuclear extracts were prepared and Egr-1 expression was analyzed via immunoblotting. Egr-1 immunoreactivity was almost undetectable in the absence of stimulation. In contrast, thrombin and carbachol strikingly increased the biosynthesis of Egr-1 with a peak expression occurring 1 hour following stimulation (Fig. 1A). The expression of Sp1 was analyzed as a loading control.

### The newly synthesized Egr-1 is biologically active

The ability of Egr-1 to activate transcription depends upon the concentrations of the Egr-1 negative cofactors NAB1 and NAB2. These proteins bind to Egr-1 and block transcriptional activation via Egr-1 [17-19]. Thus, elevated Egr-1 protein levels do not automatically indicate an increased transcription of Egr-1 target genes. We determined the transcription of an Egr-1-responsive target gene in thrombin and carbachol-treated 39M1-81 cells using a chromosomally embedded Egr-1-responsive luciferase reporter gene. The reporter gene has been integrated into the genome of the cells via lentiviral gene transfer. A schematic depiction of the integrated provirus is shown in Fig. 1B. The implanted transcription unit encoded the reporter gene luciferase, controlled by a minimal promoter consisting of four binding sites for Egr-1, derived from the human synapsin I promoter, a TATA box and an initiator element. The infected cells were stimulated with thrombin or carbachol and gene transcription of the integrated reporter was measured. The results show that treatment of 39M1-81 cells with thrombin or carbachol significantly increased the transcription of the Egr-1-responsive reporter gene (Fig. 1C), indicating that biologically active Egr-1 was synthesized.

### Role of Ca^2+^-ions in thrombin and carbachol-induced Egr-1 biosynthesis in 39M1-81 cells

**Figure 2:**
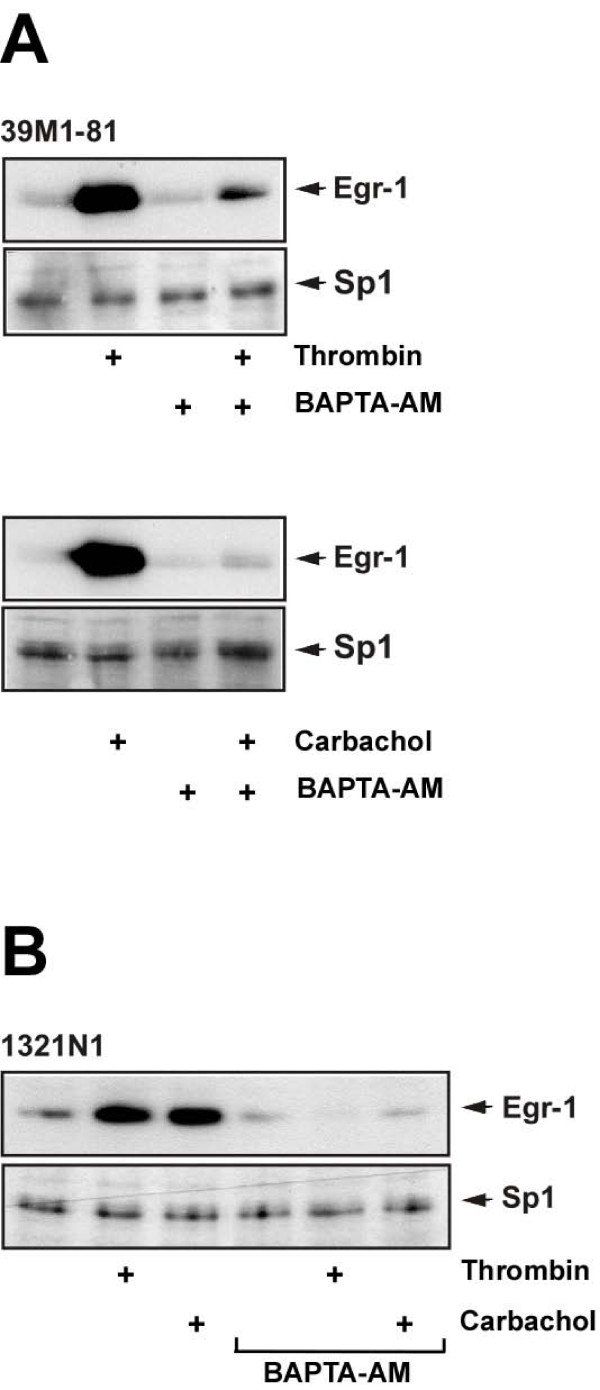
**Chelation of intracellular Ca^2+ ^attenuates expression of Egr-1 in thrombin or carbachol stimulated 39M1-81 fibroblasts and in 1321N1 glioma cells**. 39M1-81 (A) or 1321N1 (B) cells were preincubated for 30 min with the calcium chelator BAPTA-AM (50 μM). Cells were stimulated with either thrombin or carbachol for 1 hour. Nuclear extracts were prepared and subjected to Western blot analysis. The blots were incubated with an antibody directed against Egr-1.

Stimulation of protease-activated receptors and M_1 _muscarinic acetylcholine receptors leads to the activation of phospholipase C, the generation of IP_3 _and the release of Ca^2+^-ions into the cytosol via stimulation of ionotropic IP_3 _receptors of the endoplasmic reticulum. Accordingly, thrombin and carbachol increased the free cytosolic Ca^2+ ^concentration in 39M1-81 cells [9]. Therefore, we assessed the importance of elevated [Ca^2+^]_i _for the signaling cascade connecting protease-activated and muscarinic M_1 _receptor activation with Egr-1 gene transcription. 39M1-81 cells were preincubated in the presence or absence of the cell-permeable acetoxymethylester of the cytosolic Ca^2+ ^chelator BAPTA following stimulation with either thrombin or carbachol. When the thrombin or carbachol-induced elevation of [Ca^2+^]_i _was precluded by the preincubation with BAPTA-AM, the stimulus-induced biosynthesis of Egr-1 was almost completely blocked (Fig. 2A). Similar results were obtained with human 1321N1 astrocytoma cells that express protease-activated receptors and M_3 _muscarinic acetylcholine receptors [10] (Fig. 2B). Hence, elevation of cytosolic Ca^2+ ^levels is essential for induction of Egr-1 biosynthesis following thrombin and M_1 _or M_3 _muscarinic acetylcholine receptor stimulation in 39M1-81 fibroblasts or 1321N1 astrocytoma cells.

### Expression of Egr-1 in thrombin or carbachol-stimulated 39M1-81 cells requires activation of ERK

**Figure 3:**
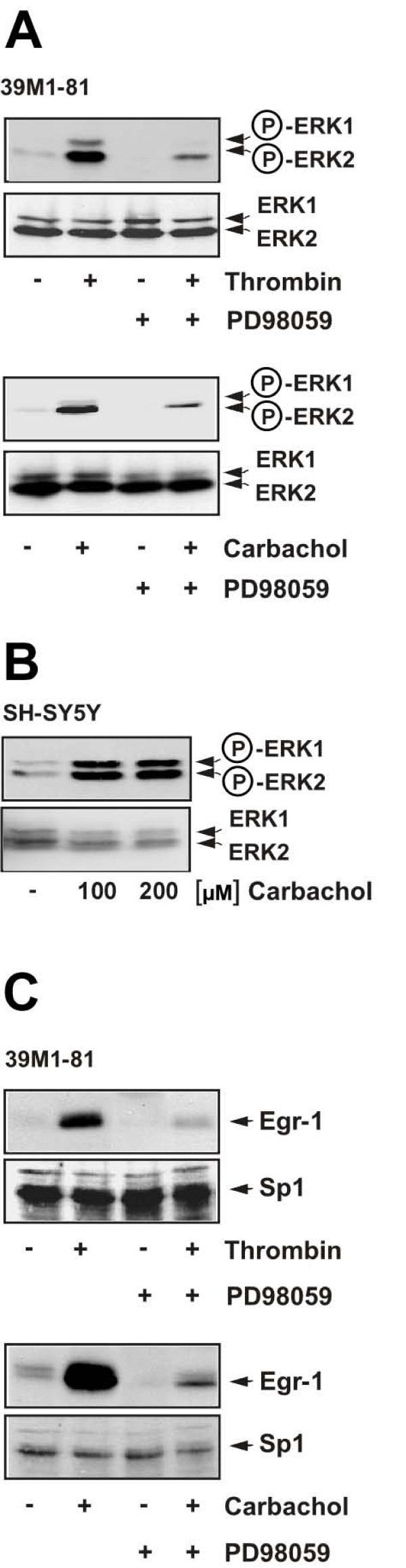
**Essential role of ERK activation in thrombin and carbachol-triggered upregulation of Egr-1 expression**. (A) 39M1-81 cells were serum-starved for 24 hours and treated with thrombin (1 U/ml) or carbachol (100 μM) for 5 min. 39M1-81 cells were preincubated for 6 hours with PD98059 (50 μM) as indicated. Whole cell extracts were prepared and subjected to Western blot analysis. The blots were incubated with a monoclonal antibody directed against the phosphorylated active forms of ERK1 and ERK2. (B) SH-SY5Y neuroblastoma cells were incubated in medium containing 0.05% serum for 24 hours and treated with carbachol (100 μM) for 15 min. Whole cell extracts were prepared and subjected to Western blot analysis. The blots were incubated with a monoclonal antibody directed against the phosphorylated active forms of ERK1 and ERK2. (C) The activation of ERK is essential to connect thrombin and carbachol stimulation enhanced Egr-1 biosynthesis. 39M1-81 cells were preincubated for 6 hours with PD98059 (50 μM). Cells were stimulated with thrombin (1 U/ml) or carbachol (100 μM) for 1 hour. Nuclear extracts were prepared and subjected to Western blot analysis. The blots were incubated with an antibody directed against Egr-1.

Stimulation of CCL39 cells with thrombin activates ERK [2,20]. Likewise, stimulation of muscarinic acetylcholine receptors has been shown to trigger the phosphorylation and activation of ERK in different cell types [11,21,22]. We used an antibody directed against the phosphorylated isoforms of ERK1 and ERK2 to analyze the effect of either thrombin or carbachol stimulation on the phosphorylation state of ERK1 and ERK2 in 39M1-81 cells. As shown in Fig. 3A, administration of either thrombin or carbachol induced the phosphorylation, i.e. activation of ERK2 in these cells. Preincubation of the cells for six hours with PD98059, a compound that inhibits phosphorylation of MAP kinase kinase, reduced stimulus-induced ERK2 phosphorylation. Higher levels of ERK2 are expressed in 39M1-81 cells in comparison to ERK1. Phosphorylation of ERK1 was also induced in thrombin and carbachol-stimulated 39M1-81 cells, but to a lesser extent (Fig. 3A). As a control, we show that ERK1 and ERK2 are phosphorylated in carbachol-treated SH-SY5Y neuroblastoma cells (Fig. 3B).

In many cellular systems the activation of the ERK signaling cascade is the major stimulus for induction of the biosynthesis of Egr-1 [15,16]. This is also the case for 39M1-81 cells, as shown in Fig. 3C. 39M1-81 cells were preincubated for six hours with PD98059. This treatment efficiently blocked the biosynthesis of Egr-1 in 39M1-81 cells that had been stimulated with either thrombin or carbachol.

### Conditional activation of the ERK signaling pathway in 39M1-81 fibroblasts via expression of a ΔRaf-1-estrogen receptor fusion protein

**Figure 4:**
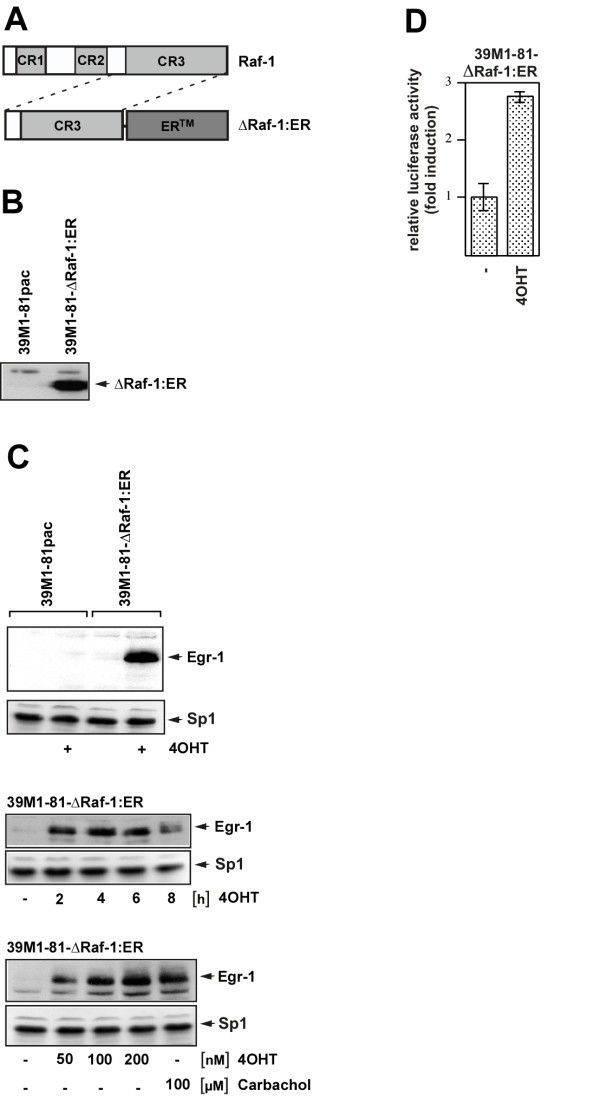
**Generation of 39M1-81 cells expressing a conditionally active form of Raf-1 protein kinase**. (A) Schematic representation of the modular structure of Raf-1 and ΔRaf-1:ER. (B) Expression of ΔRaf-1:ER in 39M1-81 cells. Whole cell extracts of ΔRaf-1:ER expressing 39M1-81 cells were prepared and analyzed by immunoblotting using an antibody directed against the murine estrogen receptor. As a control, 39M1-81 cells were analyzed expressing the selection marker puromycin acetyltransferase (pac). (C) Upper panel: 39M1-81-ΔRaf-1:ER cells and 39M1-81pac cells were stimulated with 4OHT (100 nM) for 2 hours. Nuclear extracts were prepared and subjected to Western blot analysis. The blot was incubated with an antibody directed against Egr-1. Middle panel: Sustained synthesis of Egr-1 in 4OHT-treated 39M1-81-ΔRaf-1:ER cells. 39M1-81-ΔRaf-1:ER cells were stimulated with 4OHT (100 nM) for 2, 4, 6, or 8 hours as indicated. Nuclear extracts were prepared and subjected to Western blot analysis. The blot was incubated with an antibody directed against Egr-1. Lower panel: 39M1-81-ΔRaf-1:ER cells were stimulated for 2 hours with different concentrations of 4OHT as indicated. For comparison, cells were stimulated with carbachol (100 μM). Egr-1 expression was visualized by Western blotting using an anti-Egr-1 antibody. (D) The newly synthesized Egr-1 protein in 4OHT-treated 39M1-81-ΔRaf-1:ER cells is biologically active. 39M1-81-ΔRaf-1:ER cells were infected with a recombinant lentivirus encoding an Egr-1 responsive reporter gene. The infected cells were stimulated with 4OHT (100 nM) for 16 hours. Cell extracts were prepared and analyzed for luciferase activities which were normalized to the protein concentrations.

In the previously described experiments we showed that ERK activation is a key event in the signaling cascades that connects protease-activated and M_1 _muscarinic acetylcholine receptor stimulation with Egr-1 gene transcription. To specifically activate the ERK pathway in 39M1-81 cells in a ligand-independent manner and to confirm the importance of the ERK signaling pathway for thrombin and carbachol-induced upregulation of Egr-1, we generated 39M1-81 cells expressing a ΔRaf-1-estrogen receptor fusion protein, a conditionally active form of Raf-1 protein kinase. The modular structure of Raf-1 and ΔRaf-1:ER is schematically depicted in Fig. 4A. Raf-1 contains three functional domains (CR1, CR2 and CR3). CR1 is a cysteine-rich region and functions as binding site for activated Ras (Ras-GTP) at the cell membrane. CR2 is rich in serine and threonine residues and negatively regulates the biological activity of the catalytic domain. CR3 encompasses the protein kinase domain. We expressed the catalytic domain of Raf-1 as a fusion protein with the ligand binding domain of the murine estrogen receptor (ER) to keep the protein kinase in an inactive state in the absence of hormone. Addition of hormone leads to an enhancement of Raf-1 kinase activity [23]. The use of the estrogen receptor mutant termed ER^Tamoxifen Mutant ^allowed us to use the synthetic ligand, 4-hydroxytamoxifen (4OHT) for induction. We infected 39M1-81 cells with a recombinant retrovirus encoding ΔRaf-1:ER. As a control, 39M1-81 cells were infected with a recombinant retrovirus encoding puromycin acetyltransferase. The expression of the Raf-1/estrogen receptor fusion protein that occured under control of the murine stem cell virus long terminal repeat was verified using an antibody specific for the estrogen receptor ligand binding domain (Fig. 4B). Next, 39M1-81-ΔRaf-1:ER and 39M1-81pac cells were stimulated with 4OHT for 2 hours. Fig. 4C (upper panel) shows that Egr-1 was synthesized in 39M1-81-ΔRaf-1:ER cells, but not in 39M1-81pac cells. The biosynthesis of Egr-1 was sustained and lasted at least for 6 hours (Fig. 4C, middle panel). Stimulation of 39M1-81-ΔRaf-1:ER cells with 4OHT for 2 hours induced the biosynthesis of Egr-1 to a similar level as stimulation of the cells with carbachol (Fig. 4C, lower panel). Furthermore, stimulation of 39M1-81-ΔRaf-1:ER cells with 4OHT upregulated transcription of a chromatin-embedded Egr-1-responsive reporter gene (Fig. 4D).

### Role of EGF receptor transactivation in thrombin and carbachol-induced biosynthesis of Egr-1

**Figure 5:**
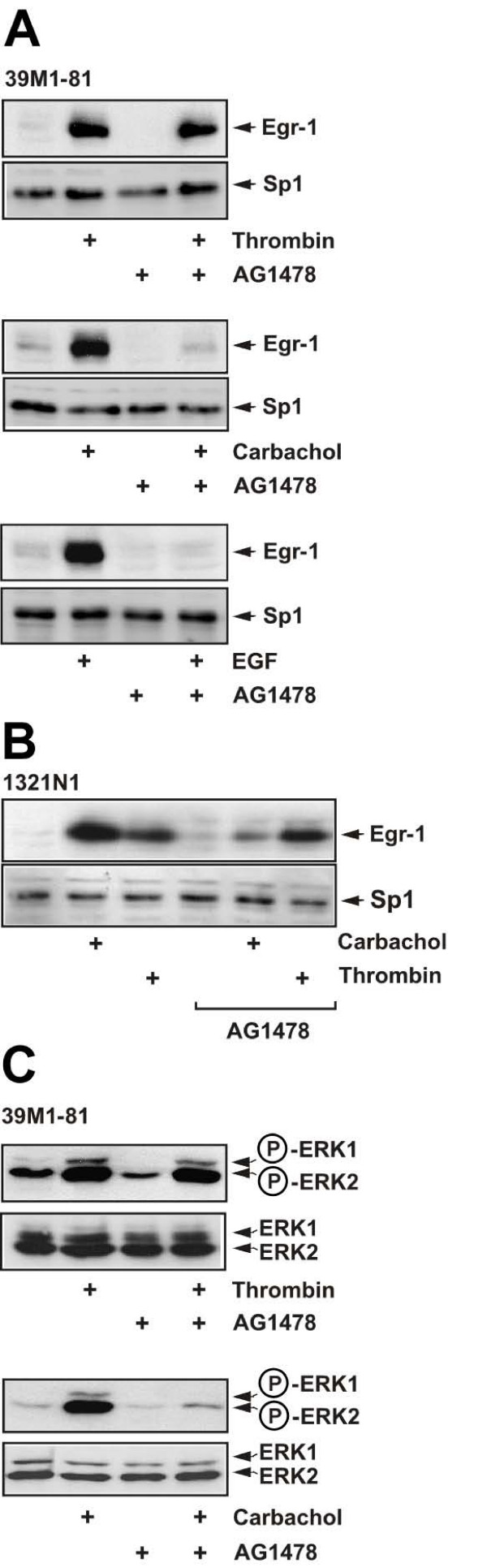
**Role of EGF receptor transactivation in thrombin signaling**. 39M1-81 cells (A) or 1321N1 glioma cells (B) were preincubated with AG1478 (0.5 μM) or vehicle and then stimulated with either thrombin (1 U/ml) or carbachol (100 μM) for 1 hour. Nuclear extracts were prepared and subjected to Western blot analysis using an antibody directed against Egr-1. As a control, cells were stimulated with EGF (10 ng/ml) in the presence or absence of AG1478 (A, lower panel). (C) 39M1-81 cells were preincubated with AG1478 (0.5 μM) or vehicle and then stimulated with either thrombin (1 U/ml) or carbachol (100 μM) for 5 min. Whole cell extracts were prepared and subjected to Western blot analysis. The blots were incubated with a monoclonal antibody directed against the phosphorylated active forms of ERK1 and ERK2.

Elevation of the intracellular Ca^2+ ^concentration may trigger transactivation of the EGF receptor and/or activation of PKC. Recently, we showed that upregulation of Egr-1 expression after stimulation of ionotropic P2X_7 _receptors requires the transactivation of the EGF receptor [24]. We therefore tested whether stimulation of 39M1-81 cells with thrombin or carbachol, resulting in an influx of Ca^2+ ^into the cells, also leads to a transactivation of the EGF receptor. We preincubated 39M1-81 cells with the tyrosine kinase inhibitor AG1478 for 1 hour before we stimulated the cells with either thrombin or carbachol. Fig. 5A (upper panel) shows that expression of Egr-1 in thrombin-stimulated 39M1-81 cells was not impaired in AG1478-pretreated cells, indicating that the signaling cascade connecting thrombin stimulation with enhanced Egr-1 gene transcription was independent of EGF receptor transactivation. In contrast, carbachol-induced upregulation of Egr-1 was blocked in cells preincubated with AG1478 (Fig. 5A, middle panel), indicating that transactivation of the EGF receptor is a necessary link for M_1 _muscarinic acetylcholine-receptor signaling in 39M1-81 cells. As a control, we showed that AG1478 totally blocked EGF signaling in 39M1-81 cells (Fig. 5A, lower panel). Similar results were obtained in thrombin and carbachol stimulated 1321N1 astrocytoma cells (Fig. 5B). These results were corroborated by an analysis of ERK phosphorylation in thrombin and carbachol-stimulated cells that had been pretreated with AG1478. Fig. 5C shows that while phosphorylation of ERK was largely reduced by AG1478 pretreatment in carbachol-stimulated 39M1-81 cells, ERK was still phosphorylated in AG1478-pretreated cells that had been stimulated with thrombin.

### PKC connects protease-activated and muscarinic acetylcholine receptor activation with ERK phosphorylation and Egr-1 biosynthesis

**Figure 6:**
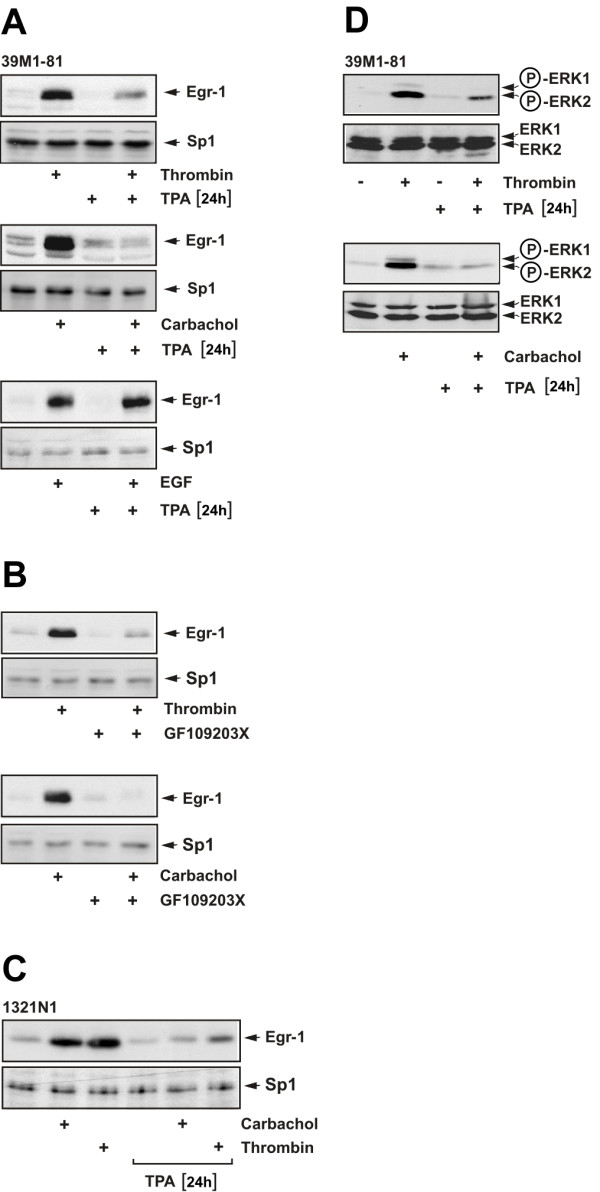
**Thrombin-regulated expression of Egr-1 requires the activation of PKC**. 39M1-81 cells (A) or 1321N1 astrocytoma cells (C) were preincubated with TPA (100 ng/ml) or vehicle for twenty-four hours and then stimulated with either thrombin (1 U/ml) or carbachol (100 μM) for 1 hour. Nuclear extracts were prepared and subjected to Western blot analysis using an antibody directed against Egr-1. As a control, cells were stimulated with EGF (10 ng/ml) in the presence or absence of TPA (A, lower panel). (B) 39M1-81 cells were preincubated with the PKC inhibitor GF109203X (10 μM) or vehicle for 30 min and then stimulated with either thrombin (1 U/ml) or carbachol (100 μM) for 1 hour. Nuclear extracts were prepared and subjected to Western blot analysis using an antibody directed against Egr-1. (D) 39M1-81 cells were preincubated with TPA (100 ng/ml) or vehicle for twenty-four hours and then stimulated with either thrombin (1 U/ml) or carbachol (100 μM) for 5 min. Whole cell extracts were prepared and subjected to Western blot analysis. The blots were incubated with a monoclonal antibody directed against the phosphorylated active forms of ERK1 and ERK2.

The fact that transactivation of the EGF receptor is not required to connect thrombin stimulation of 39M1-81 cells with enhanced Egr-1 expression let us to assessing the role of PKC in thrombin-induced signaling. In gonadotropin-releasing hormone stimulated gonadotrophs, Egr-1 biosynthesis occurs via activation of PKC [25]. Likewise, thrombin stimulation has been shown to activate PKC in rat astrocytes [26]. Therefore, 39M1-81 fibroblasts and 1321N1 astrocytoma cells were incubated with the phorbol ester TPA for twenty-four hours to downregulate diacylgycerol-regulated protein kinase C isoforms by promoting proteolytic degradation as described [9]. Fig. 6A reveals that prolonged treatment with TPA attenuated the thrombin and carbachol-induced signaling pathway leading to the biosynthesis of Egr-1. EGF is known to trigger an upregulation of Egr-1 in 39M1-81 cells, as depicted in Fig. 5A (lower panel). However, activation of protein kinase C was not required for EGF-induced upregulation of Egr-1 expression (Fig. 6A, lower panel). Preincubation of 39M1-81 cells with the PKC inhibitor GF109203X blocked the biosynthesis of Egr-1 in thrombin or carbachol-stimulated cells (Fig. 6B), supporting the conclusion that PKC activation is required to connect stimulation of the cells with enhanced Egr-1 biosynthesis. Downregulation of diacylgycerol-regulated protein kinase C isoforms also impaired thrombin and carbachol-elicited expression of Egr-1 in 1321N1 astrocytoma cells (Fig. 6C). In addition, prolonged treatment with TPA blocked the phosphorylation of ERK1/2 in 39M1-81 cells that had been stimulated with either thrombin or carbachol (Fig. 6D). These results underline the importance of PKC activation for connecting receptor stimulation with enhanced Egr-1 gene transcription and further supports the previous conclusion that phosphorylation and activation of ERK is essential for connecting thrombin or carbachol stimulation with enhanced Egr-1 biosynthesis.

### Thrombin-response elements are present in the human Egr-1 promoter

**Figure 7:**
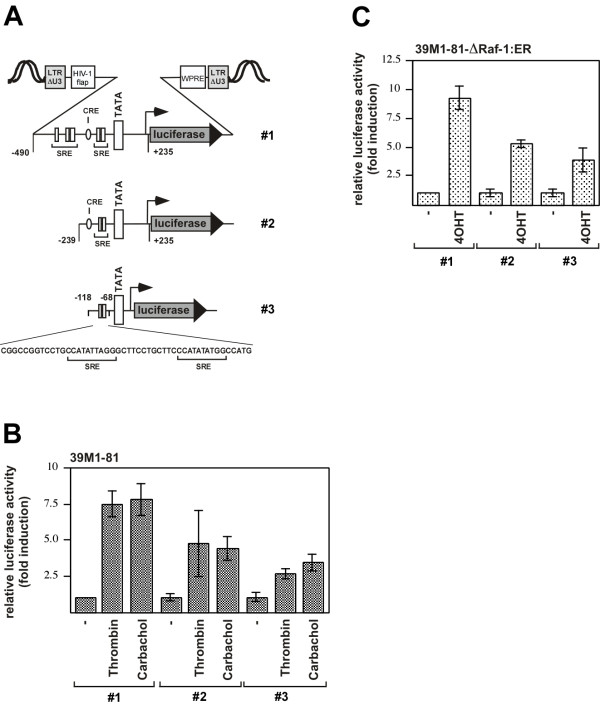
**Transcriptional upregulation of Egr-1 promoter/luciferase reporter gene transcription in thrombin or carbachol stimulated 39M1-81 cells**. (A) Schematic representation of integrated proviruses encoding Egr-1 promoter/luciferase reporter genes. The transfer vectors pFWEgr1.2luc (# 1) and pFWEgr-1.1luc (# 2) contain the sequences from -490 to + 235 or from -239 to +235 derived from the human Egr-1 gene. The transfer vector pFWEgr-1SREluc (# 3) contains the two proximal SREs # 1 and 2 of the Egr-1 promoter upstream of a minimal promoter. The important genetic elements within the Egr-1 regulatory region are depicted, including five serum response elements (SRE), and a cAMP response element (CRE). The U3 region of the 5' LTR of the transfer vector is deleted. The woodchuck hepatitis virus posttranscriptional regulatory element (WPRE) and the HIV flap element are indicated. (B, C) 39M1-81 cells (B) or 39M1-81-ΔRaf-1:ER cells (C) were infected with recombinant lentiviruses generated with the transfer vectors pFWEgr-1.1luc, pFWEgr-1.2luc, or pFWEgr-1SREluc. The infected cells were treated with thrombin, carbachol, or 4OHT for sixteen hours. Cell extracts were prepared and analyzed for luciferase activities. Luciferase activity was normalized to the protein concentration. Data are means ± SD for n = 4 experiments.

To identify genetic elements that mediate thrombin-responsiveness of the Egr-1 gene we inserted Egr-1 promoter/luciferase reporter genes into the chromatin of 39M1-81 cells using lentiviral gene transfer. The 5'-flanking region of the Egr-1 gene contains five serum response elements, and these motifs are responsible for the induction of Egr-1 gene transcription by various extracellular signaling molecules [15,16]. The transfer vectors pFWEgr-1.1luc (# 2) and pFWEgr-1.2luc (#1), used to generate recombinant lentiviruses, encode Egr-1 promoter/luciferase reporter genes that contained 239 or 490 nucleotides of the human Egr-1 gene 5' upstream region, respectively, together with 235 nucleotides of the 5'-nontranslated region. Lentiviruses generated with the transfer vector pFWEgr-1SREluc (#3) encoded for the luciferase reporter gene controlled by a minimal promoter and the proximal SREs of the Egr-1 promoter. Fig. 7A shows a schematic depiction of the integrated proviruses encoding Egr-1 promoter/luciferase reporter genes. 39M1-81 cells were infected with recombinant lentiviruses and stimulated. The addition of either thrombin or carbachol increase transcription of a reporter gene ~7.5 fold or 7.8-fold, that was controlled by 490 nucleotides of the human Egr-1 promoter (Fig. 7B, #1, left panels). Transcription of the reporter gene controlled by 239 nucleotides of the Egr-1 regulatory region (# 2) was increased by 4.7-fold (thrombin) or 4.4-fold (carbachol) (Fig. 7B, middle panels). Stimulation of a reporter gene controlled by the proximal SREs of the Egr-1 promoter (# 3) led to a 2.6-fold (thrombin) or 3.4-fold stimulation of reporter gene transcription (Fig. 7B, right panels). Thus, both proximal and distal SREs contributed to an enhanced reporter gene transcription in stimulated 39M1-81 cells. Likewise, luciferase expression was enhanced in 4OHT-treated 39M1-81-ΔRaf-1:ER cells that has been infected with lentiviruses expressing the indicated reporter genes (Fig. 7C). Incubation of the cells with 4OHT induced a 9.2-fold (# 1), 5.2-fold (# 2), or a 3.8-fold elevation of reporter gene transcription in these cells.

### Elk-1 is phosphorylated in thrombin or carbachol-stimulated 39M1-81 cells

**Figure 8:**
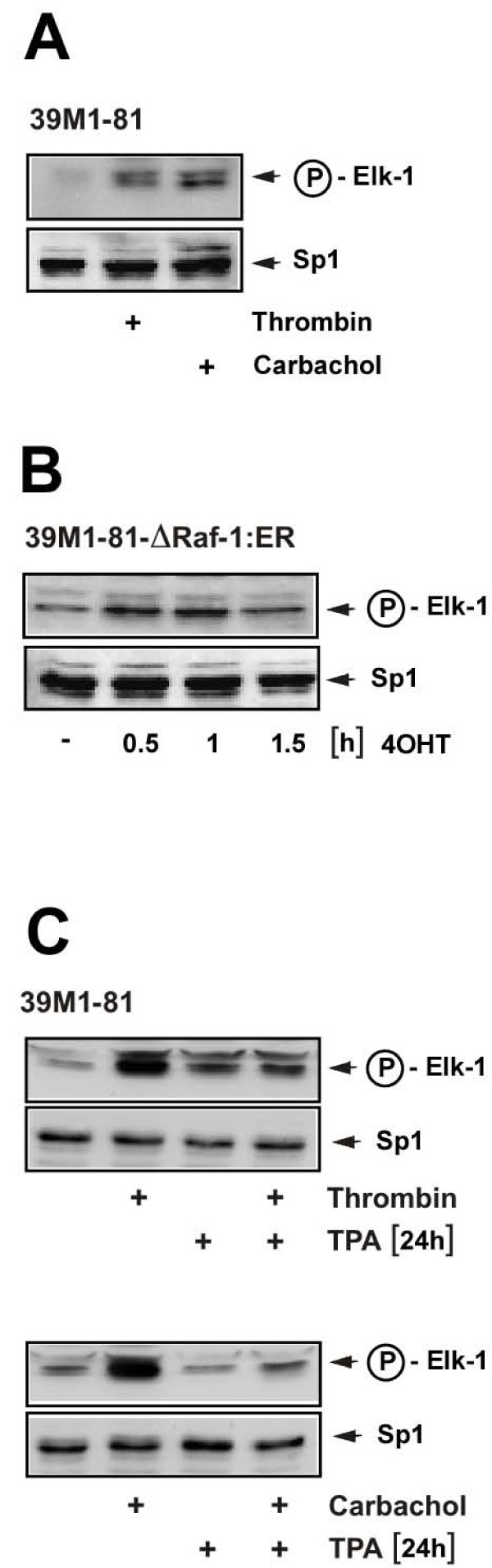
**Phosphorylation of Elk-1 in thrombin and carbachol-stimulated 39M1-81 cells**. (A) 39M1-81 cells were stimulated with thrombin or carbachol for 15 min. Nuclear extracts were prepared and subjected to Western blot analysis using a phospho-specific monoclonal antibody that detected phosphorylation of Elk-1 on serine residue 383. As a control, the expression of Sp1 was assessed. (B) 39M1-81-ΔRaf-1:ER cells were stimulated with 4OHT for 0.5, 1, or 1.5 hours. Nuclear extracts were prepared and subjected to Western blot analysis using the phospho-specific anti-Elk-1 antibody. As a control, the expression of Sp1 was assessed. (C) 39M1-81 cells were preincubated with TPA (100 ng/ml) or vehicle for twenty-four hours and then stimulated with either thrombin (1 U/ml) or carbachol (100 μM) for 15 min. Nuclear extracts were prepared and subjected to Western blot analysis using a monoclonal antibody directed against the phosphorylated form of Elk-1.

Phosphorylated ERK translocates into the nucleus, where it is able to change the transcriptional program by phosphorylation of transcriptional regulatory proteins. Major ERK substrates in the nucleus are ternary complex factors such as Elk-1, key transcription factors of serum response element-mediated gene transcription. Fig. 8A shows that Elk-1 was phosphorylated at serine residue 383 in 39M1-81 cells after stimulation with either thrombin or carbachol. In addition, stimulation of 39M1-81-ΔRaf-1:ER cells with 4OHT triggered the phosphorylation of Elk-1 (Fig. 8B), indicating that activation of the ERK signaling pathway is sufficient to induce phosphorylation of Elk-1. Elk-1 was already phosphorylated to a lesser extent in unstimulated cells. Thrombin and carbachol-induced phosphorylation of Elk-1 was impaired in cells preincubated for twenty-four hours with TPA, to downregulate diacylgycerol-regulated protein kinase C isoforms (Fig. 8C). These data indicate that activation of PKC is necessary to induce Elk-1 phosphorylation in thrombin and carbachol stimulated 39M1-81 cells.

### Suppression of ternary complex factor activity impairs the upregulation of Egr-1 biosynthesis in thrombin or carbachol-stimulated fibroblasts

**Figure 9:**
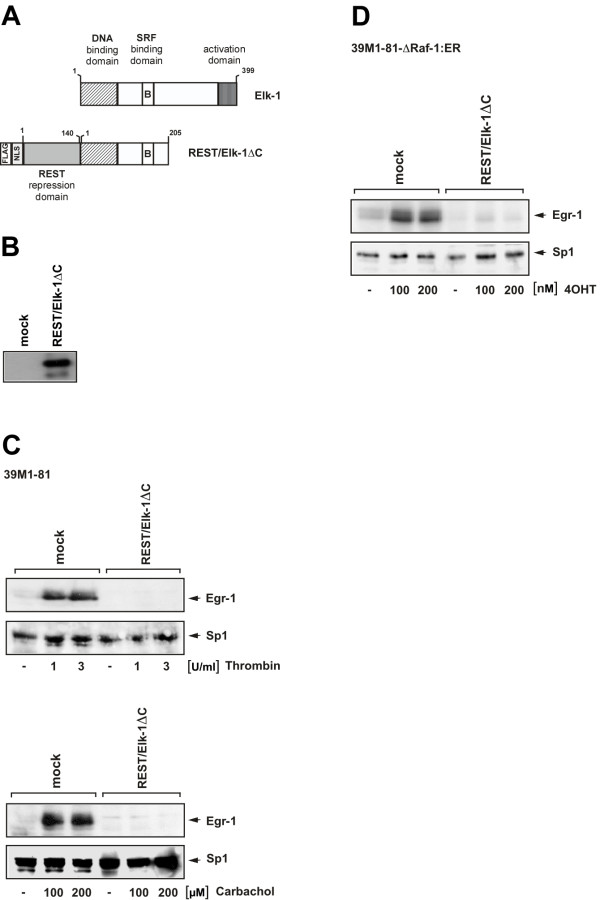
**Essential role of ternary complex factor activation in the regulation of thrombin and carbachol-induced Egr-1 biosynthesis in 39M1-81 cells**. (A) Schematic representation of wild-type Elk-1 and the dominant-negative mutant REST/Elk-1ΔC. (B) Western blot analysis of 39M1-81 cells either mock infected or infected with a recombinant lentivirus encoding REST/Elk-1ΔC. The Western blot was probed with an antibody against the FLAG-tag. (C) Expression of REST/Elk-1ΔC blocks thrombin and carbachol-induced upregulation of Egr-1 in 39M1-81 cells. 39M1-81 cells were either mock infected or infected with a recombinant lentivirus encoding REST/Elk-1ΔC. Cells were stimulated with either thrombin or carbachol as indicated. Nuclear extracts were prepared and subjected to Western blot analysis. The blot was incubated with an antibody directed against Egr-1. As a control, the expression of Sp1 was determined in mock and virus infected cells. (D) Expression of REST/Elk-1ΔC blocks 4OHT-induced upregulation of Egr-1 in 39M1-81-ΔRaf-1:ER cells. 39M1-81 cells were either mock infected or infected with a recombinant lentivirus encoding REST/Elk-1ΔC. Cells were stimulated with 4OHT as indicated. Nuclear extracts were prepared and subjected to Western blot analysis. The blot was incubated with an antibody directed against Egr-1. As a control, the expression of Sp1 was determined in mock and virus infected cells.

Given the importance of SREs within the Egr-1 promoter, we next assessed the impact of ternary complex factor activation on the regulation of Egr-1 biosynthesis in thrombin or carbachol-stimulated 39M1-81 cells. To overcome the problem associated with redundancy of functions between the ternary complex factors, we expressed a dominant-negative mutant of the ternary complex factor Elk-1, termed REST/Elk-1ΔC. The mutant retains the DNA-binding and SRF interaction domain, but lacks the C-terminal activation domain of Elk-1 (Fig. 9A). REST/Elk-1ΔC additionally contains the N-terminal repression domain of the transcriptional repressor REST [27], an immunological tag used for detection of the protein (FLAG epitope) and a nuclear localization signal (NLS). Nuclear proteins of mock-infected 39M1-81 cells or cells infected with a REST/Elk-1ΔC encoding lentivirus were fractionated by SDS-PAGE. The fusion protein was identified by Western blot analysis using antibodies targeting the FLAG epitope. Fig. 9B shows that the REST/Elk-1ΔC fusion protein was synthesized as expected. Next, the functional implication of REST/Elk-1ΔC expression were assessed. The results show that REST/Elk-1ΔC blocked the biosynthesis of Egr-1 in thrombin and carbachol-stimulated 39M1-81 cells. In contrast, the expression of Sp1 was not changed in mock infected cells in comparison to cell infected with a REST/Elk-1ΔC encoding lentivirus (Fig. 9C).

### Suppression of ternary complex factor activity impairs the upregulation of Egr-1 biosynthesis in 4OHT-stimulated 39M1-81-ΔRaf-1:ER cells

In 39M1-81-ΔRaf-1:ER cells, the biosynthesis of Egr-1 is induced after stimulation of the ERK signaling pathway by 4OHT. Fig. 9D reveals that the connection between the activated ERK signaling pathway and the transcription of the Egr-1 gene is interrupted in cells that express the Elk-1 mutant REST/Elk-1ΔC. This results further supports the view that phosphorylation and activation of ternary complex factors is a key step in connecting intracellular signaling with Egr-1 gene transcription.

### Suppression of ternary complex factor activity blocks the transcription of SRE-regulated reporter gene

**Figure 10:**
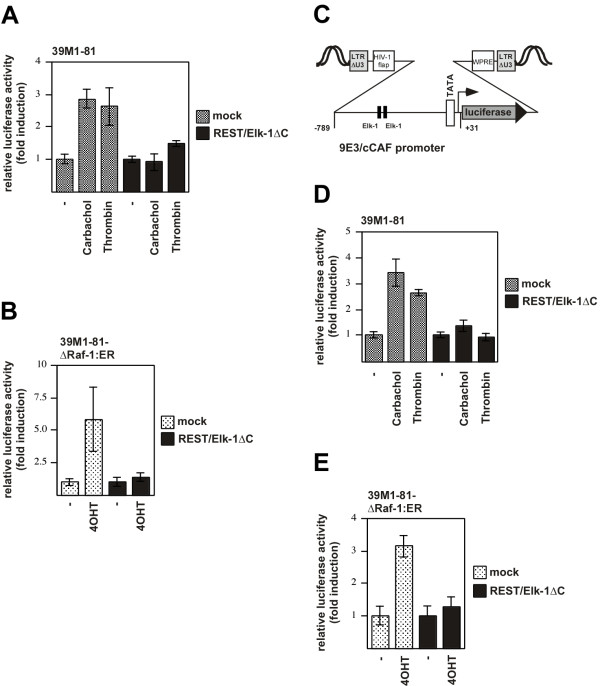
**Expression of a dominant-negative mutant of Elk-1 blocks transcription of Elk-1-regulated reporter genes**. 39M1-81 cells (A) or 39M1-81-ΔRaf-1:ER cells (B) were infected with a recombinant lentivirus encoding a luciferase reporter gene controlled by the proximal SREs of the Egr-1 promoter. 72 hours later, cells were either mock infected or infected with a lentivirus encoding REST/Elk-1ΔC. Cells were stimulated with either thrombin (1 U/ml) or carbachol (100 μM) (A) or 4OHT (100 nM) (B) for 16 hours. Cells were harvested, cell extracts were prepared and analyzed for luciferase activities. Luciferase activity was normalized to the protein concentration. (C) Schematic representation of an integrated provirus encoding a 9E3/cCAF promoter/luciferase reporter gene. The transfer vector pFW9E3luc contains the sequences from -789 to +31 derived from the chicken 9E3/cCAF gene. The Elk-1 binding sites within the 9E3/cCAF regulatory region are depicted. (D, E) 39M1-81 cells (D) or 39M1-81-ΔRaf-1:ER cells (E) were infected with a recombinant lentivirus encoding a luciferase reporter gene controlled by the 9E3/cCAF promoter. 72 hours later, cells were either mock infected or infected with a lentivirus encoding REST/Elk-1ΔC. Cells were stimulated with either thrombin (1 U/ml) or carbachol (100 μM) (D) or 4OHT (100 nM) (E) for 16 hours. Cells were harvested, cell extracts were prepared and analyzed for luciferase activities. Luciferase activity was normalized to the protein concentration.

The Elk-1 mutant REST/Elk-1ΔC binds to DNA and to the SRF and thus blocks the access of wild-type Elk-1 to its cognate DNA binding sites. We directly assessed the effect of REST/Elk-1ΔC on gene transcription by performing double-infection experiments. Cells were first infected with a lentivirus encoding the luciferase reporter gene under control of the proximal SREs derived from the Egr-1 promoter (#3 in Fig. 7A). 72 hours later, we infected the cells with a lentivirus encoding REST/Elk-1ΔC. As a control, mock-infected cells were analyzed. Fig. 10A shows that expression of REST/Elk-1ΔC reduced or abolished upregulation of reporter gene transcription in thrombin or carbachol-stimulated 39M1-81 cells. Likewise, reporter gene transcription was blocked in 4OHT-stimulated 39M1-81-ΔRaf-1:ER cells that expressed REST/Elk-1ΔC (Fig. 10B)

### Expression of a dominant-negative Elk-1 mutant suppresses thrombin and carbachol-induced upregulation of 9E3/cCAF promoter activity

The previous experiments indicated that ternary complex factor activation links the thrombin-induced signaling cascade with the Egr-1 gene. Expression of the chemokine 9E3/cCAF has also been shown to be induced after thrombin stimulation. A molecular dissection of the signaling pathway revealed that Elk-1 activates 9E3/cCAF expression via two Elk-1 binding sites in the proximal promoter region [28]. We therefore analyzed the regulation of a reporter gene that was controlled by the 9E3/cCAF promoter. 39M1-81 fibroblasts were infected with a lentivirus that encoded for the luciferase reporter gene, controlled by the 9E3/cCAF promoter. A schematic representation of the integrated provirus is depicted in Fig. 10C. 39M1-81 cells were infected with a lentivirus encoding the luciferase reporter gene under control of the 9E3/cCAF promoter. Treatment of the cells with thrombin or carbachol stimulated transcription of the reporter gene (Fig. 10D). Likewise, reporter gene transcription was enhanced in 4OHT-stimulated 39M1-81-ΔRaf-1:ER cells (Fig. 10E). Expression of REST/Elk-1ΔC abolished stimulus-induced reporter gene transcription (Figs. 10D, E), confirming that Elk-1 is required for the stimulus-induced increase of 9E3/cCAF promoter activity.

### Stimulation of 39M1-81 cells with thrombin upregulates MKP-1 expression

**Figure 11:**
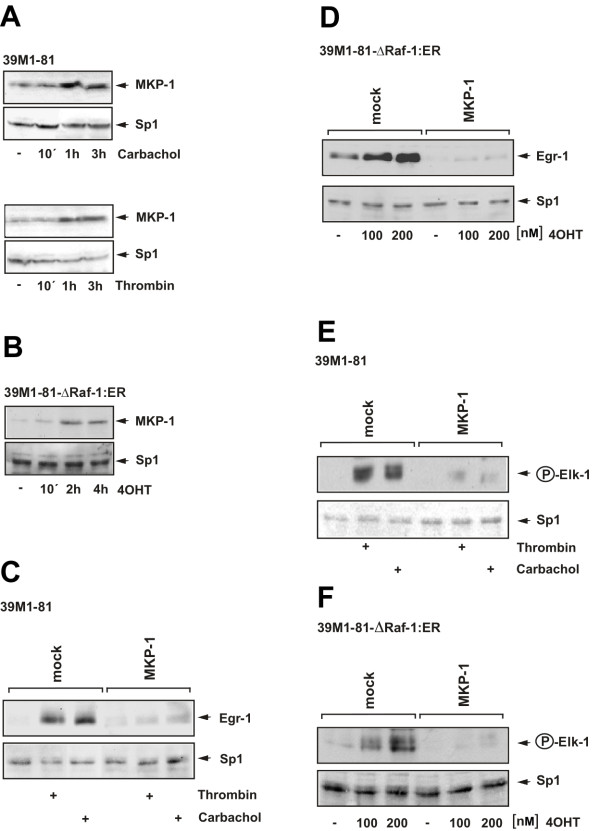
**MKP-1 overexpression prevents Egr-1 expression and Elk-1 phosphorylation in thrombin and carbachol-treated 39M1-81 cells**. (A) Western Blot analysis of 39M1-81 cells treated with either thrombin (1 U/ml) or carbachol (100 μM). Nuclear extracts were prepared and subjected to Western blot analysis using an antibody directed against MKP-1. As a control expression of Sp1 is shown. (B) 39M1-81-ΔRaf-1:ER cells were stimulated with 4OHT (100 nM). Western blot analysis of nuclear proteins was performed with an antibody directed against MKP-1. (C) Mock-infected cells or cells infected with a MKP-1 encoding lentivirus were serum-starved for twenty-four hours and then treated with either thrombin (1 U/ml) or carbachol (100 μM) for 1 hour. Nuclear extracts were prepared subjected to Western blot analysis using an antibody directed against Egr-1. (D) MKP-1 controls expression of Egr-1 in 4OHT-stimulated 39M1-81-ΔRaf-1:ER cells. The cells were either mock-infected or infected with a recombinant lentivirus encoding MKP-1. The cells were serum-starved for twenty-four hours and then treated with 4OHT (100 nM) for 2 hours. Nuclear extracts were prepared subjected to Western blot analysis using an antibody directed against Egr-1. (E, F) Mock-infected or cells infected with a lentivirus encoding MKP-1 were serum-starved for twenty-four hours and then treated either with thrombin (1 U/ml) or carbachol (100 μM) for 15 min (E) or with 4OHT (100 nM) for 30 min (F). Nuclear extracts were prepared and subjected to Western blot analysis using an antibody directed the phosphorylated form of Elk-1.

MKP-1, the enzyme that dephosphorylates ERK in the nucleus, is synthesized in different cell types following ERK activation, suggesting that MKP-1 is part of a negative feedback loop that inactivates ERK [22,29]. In CCL39 cells MKP-1 has been shown to be synthesized after serum addition [30]. ERK activation was found to be a key step in the signaling cascade leading to Egr-1 gene transcription in thrombin-stimulated 39M1-81 cells, as shown in Fig. 3. We therefore tested whether MKP-1 is synthesized in 39M1-81 cells as a result of thrombin stimulation. As a control, we stimulated the cells with carbachol. Fig. 11A shows that thrombin and carbachol treatment of 39M1-81 cells stimulated the biosynthesis of MKP-1. MKP-1 levels remained elevated for at least 3 hours, in contrast to the rapid disappearance of Egr-1. Expression of MKP-1 was also upregulated in 4OHT-stimulated 39M1-81-ΔRaf-1:ER cells (Fig. 11B).

### Overexpression of MKP-1 prevents thrombin-induced Egr-1 expression

The functional implication of MKP-1 expression on thrombin and carbachol-induced biosynthesis of Egr-1 in 39M1-81 cells was assessed. Fig. 11C shows that both thrombin and carbachol-induced Egr-1 expression was impaired in 39M1-81 cells infected with a MKP-1-encoding lentivirus. In contrast, expression of the transcription factor Sp1 was detectable in mock and virus-infected cells to a similar degree. Expression of MKP-1 also abolished the upregulation of Egr-1 expression in 4OHT-stimulated 39M1-81-ΔRaf-1:ER cells (Fig. 11D).

### Overexpression of MKP-1 prevents the phosphorylation of Elk-1 in stimulated fibroblasts

Stimulation of 39M1-81 or 39M1-81-ΔRaf-1:ER cells with either thrombin, carbachol or 4OHT induced the activation of ERK and the subsequent phosphorylation of Elk-1. Fig. 11E shows that expression of MKP-1 prevented the phosphorylation of Elk-1 in thrombin and carbachol-stimulated 39M1-81 cells. Likewise, MPK-1 expression prevented the phosphorylation of Elk-1 in 39M1-81-ΔRaf-1:ER cells that had been stimulated with 4OHT (Fig. 11F).

## Discussion

The execution of a specific pattern of gene expression requires communication between extracellular signals and the nucleus. The objective of this study was to analyze the signaling cascade leading to the expression of the transcription factor Egr-1 in 39M1-81 cells following stimulation with thrombin. 39M1-81 cells are derived from CCL39 cells that have been frequently used as a model to study thrombin induced signal transduction. The biosynthesis of the zinc finger transcription factor Egr-1 is induced by various growth factors, hormones, and neurotransmitters [15,16], indicating that the Egr-1 gene is a convergence point for many intracellular signaling cascades. In keratinocytes, thrombin stimulation leads to an upregulation of Egr-1 expression [4]. Here, we show that Egr-1 expression is strikingly induced in 39M1-81 cells that were stimulated with thrombin. We also demonstrate that the newly synthesized Egr-1 protein was biologically active, using a lentivirus-based technique to implant an Egr-1 responsive reporter gene into the chromatin of 39M1-81 cells.

Recently, we showed that stimulation of 293 cells expressing P2X_7 _receptors with the ligand BzATP induced a transient expression of Egr-1 [24]. P2X_7 _receptors are ionotropic receptors that are permeable for small cations following stimulation. In particular, stimulation of P2X_7 _receptors leads to an influx of extracellular Ca^2+ ^ions into the cytosol [24]. Experiments using the acetoxymethylester of the cytosolic Ca^2+ ^chelator BAPTA revealed that the rise of cytosolic Ca^2+ ^concentration is essential for induction of Egr-1 biosynthesis following P2X_7 _receptor stimulation. Likewise, an increase of [Ca^2+^]_i _is required in carbachol-stimulated neuroblastoma cells and gonadotrophs that express M_3 _muscarinic acetylcholine receptors to induce the biosynthesis of Egr-1 [22,29]. In 39M1-81 cells, thrombin stimulation increased the free cytosolic Ca^2+ ^concentration [9] and we have shown here that an increase of the intracellular Ca^2+^-concentration is essential for the upregulation of Egr-1 gene transcription in thrombin stimulated 39M1-81 cells. Egr-1 is thus a Ca^2+^-ion regulated transcription factor – similar to CREB, NFAT, NF-κB and others.

An elevation of the intracellular Ca^2+ ^concentration may trigger an activation of ERK, either via activation of PKC or via the transactivation of the EGF receptor. The thrombin-activated receptors PAR-1, PAR-2, and PAR-3 belong to the G-protein coupled receptor superfamily [6,8]. Stimulation of G-protein coupled receptors can induce a transactivation of the EGF receptor [31,32]. In human glioma cells, for example, we have shown that stimulation of the G-protein coupled neurokinin receptor-1 by substance P induces the biosynthesis of Egr-1 via the transactivation of the EGF receptor [33]. Likewise, we have shown that P2X_7 _receptor stimulation induces an activation of the EGF receptor tyrosine kinase and impairment of this kinase activity interrupts the connection between receptor stimulation and enhanced Egr-1 biosynthesis [24]. The results presented here show that transactivation of the EGF receptor is necessary in 39M1-81 fibroblasts to connect M_1 _muscarinic acetylcholine receptor activation with transcription of the Egr-1 gene. In contrast, thrombin-induced signaling in 39M1-81 fibroblasts and 1321N1 astrocytoma cells is independent of EGF receptor transactivation and rather requires the activation of PKC to connect the elevation of the intracellular Ca^2+ ^concentration with an activation of the ERK signaling pathway. These results are in contrast to previously published observation showing that in HaCaT keratinocytes, transactivation of the EGF receptor is an essential event in the thrombin induced signaling cascade [4]. These results are in line with previous observations showing that EGF receptor transactivation is not necessary for thrombin-induced phosphorylation of ERK in astrocytes [34]. Thus, cell type-specific variations in the thrombin-elicited signaling cascade are obvious. We further showed that the activation of ERK is of major importance for the signaling cascade that connects thrombin and carbachol stimulation with enhanced Egr-1 gene transcription. These data were corroborated with a cell line where Egr-1 expression was induced following activation of ΔRaf-1:ER, a Raf-1/estrogen receptor fusion protein that specifically activated the ERK signaling pathway.

A major nuclear substrate for ERK is Elk-1, a member of the Ets family of transcription factors. Elk-1 is an essential component of the serum response ternary complex that binds to DNA and to the serum response factor SRF. The transcriptional activity of Elk-1 depends on its phosphorylation-status. Elk-1 is phosphorylated by several protein kinases including ERK, leading to enhanced DNA binding, ternary complex formation and SRE-mediated transcription [35]. Phosphorylation of Elk-1 connects the ERK signaling cascade with SRE-mediated gene transcription. The human Egr-1 promoter contains five SREs encompassing the consensus sequence CC [A/T]_6_GG, known as the CArG box. The SREs occur in two clusters in the Egr-1 promoter, a distal 5' cluster of 3 SREs and a proximal 3' cluster of 2 SREs. In addition, multiple binding sites for Elk-1 and other ternary complex factors are adjacent to the CArG boxes having the Ets consensus core sequence GGAA/T. Transcriptional activation of Egr-1 is often preceded by an activation of Elk-1, indicating that the SREs within the Egr-1 promoter mediate signal-induced activation of Egr-1 gene transcription. For example, in stimulated glutamatergic corticostriatal neurons a strict spatiotemporal connection between Elk-1 activation and Egr-1 mRNA synthesis has been demonstrated [36]. The analysis of Egr-1 promoter/luciferase reporter genes containing two or five SREs revealed that both SRE clusters connect thrombin stimulation with enhanced Egr-1 gene transcription. These data are in line with an earlier observation that both the distal as well as the proximal SRE clusters couple enhanced ERK activity with transcriptional upregulation [37]. Similar results were obtained in 4OHT-treated 39M1-81-ΔRaf-1:ER cells that has been infected with lentiviruses expressing the indicated reporter genes. In contrast, in primary human endothelial cells, only the proximal serum response elements are required to increase Egr-1 promoter/luciferase reporter gene transcription after thrombin stimulation [14]. Thus, our data are clearly different from those reported by these authors. The differences may rely on the analysis of different cell types (endothelial cells or fibroblasts). Alternatively, instead of using transient transfection of reporter plasmids, we used a lentivirus-based technique to implant reporter genes into the chromatin of 39M1-81 cells. The transient transfection of promoter/reporter gene containing plasmids has the disadvantage that the structure of these plasmids may be incompletely organized in comparison to cellular chromatin, and may thus resemble a prokaryotic gene organisation including a nonrestrictive transcriptional ground state. In contrast, the chromatin structure in eukaryotes causes a restrictive ground state, occluding proteins such as RNA polymerases and transcriptional regulators from binding to DNA. Hence, promoter/reporter genes should be integrated into the chromatin to investigate transcriptional regulatory mechanisms. This strategy enabled us to analyse gene transcription of reporter genes that are packed into an ordered chromatin structure.

We have shown that stimulation of 39M1-81 cells with thrombin induces the phosphorylation of Elk-1 at serine residue 383. We then went a step further and performed a loss-of-function experiment to unequivocally prove the key role of ternary complex factor activation for thrombin-induced upregulation of Egr-1 expression. Genetic inactivation of Elk-1 or other ternary complex factors in transgenic mice revealed minimal changes of the phenotype [38-40], suggesting that functional redundancy may exist. Therefore, we have assessed the necessity of ternary complex factor activation for thrombin or carbachol-induced Egr-1 biosynthesis by using a dominant negative version of Elk-1 in loss-of-function experiments. Due to its binding to DNA and SRF, the Elk-1 mutant REST/Elk-1ΔC most likely also inhibits the activity of two other ternary complex factors, SAP-1 and SAP-2. These experiments revealed that expression of REST/Elk-1ΔC almost completely blocked the stimulus-induced biosynthesis of Egr-1. Thus, ternary complex factor activation is a key step in connecting thrombin or carbachol stimulation with enhanced Egr-1 biosynthesis.

Using chromatin-integrated reporter genes, controlled by the proximal SREs from the Egr-1 promoter, we directly showed that expression of the dominant-negative mutant of Elk-1 impaired reporter gene transcription after stimulation of the cells with either thrombin or carbachol. As a control, we have analyzed the regulation of the 9E3/cCAF gene. Two Elk-1 binding sites were identified between -534 and -483 of the 9E3/cCAF promotor that functions as thrombin response elements [28]. Our data show that stimulation of 39M1-81 fibroblasts with either thrombin or carbachol induced transcription of a chromatin-embedded 9E3/cCAF promotor/luciferase reporter gene. This stimulus-induced upregulation of reporter gene expression was impaired in cells that expressed the dominant-negative Elk-1 mutant REST/Elk-1ΔC. These data confirm previous studies showing that Elk-1 is essential for the thrombin-induced expression of the 9E3/cCAF gene [28,41]. While the Egr-1 promoter contains five SREs and multiple TCF binding sites, the 9E3/cCAF promoter does not contain a SRE. Thus, the ability of Elk-1 to stimulate transcription does not require the presence of SREs. The experiments presented here show that the dominant-negative mutant REST/Elk-1ΔC blocks Elk-1-regulated gene transcription regardless of the presence or absence of SREs in the regulatory region of Elk-1-controlled genes. Finally, we showed that an impairment of Elk-1 phosphorylation by overexpression of MKP-1 also prevented Egr-1 expression in thrombin or carbachol-stimulated 39M1-81 cells. Together, these data indicate that phosphorylation and activation of Elk-1 is required to connect the ERK signaling cascade with transcription of the Egr-1 gene in thrombin or carbachol-stimulated 39M1-81 cells.

39M1-81 cells express both protease-activated receptors and M_1 _muscarinic acetylcholine receptors. The data presented here demonstrate that the signaling cascades induced by either thrombin or carbachol stimulation of 39M1-81 cells are indistinguishable, except that activation of M_1 _muscarinic acetylcholine receptors induces the transactivation of the EGF receptor. Analyzing two different cell lines, 39M1-81 fibroblasts and 1321N1 glioma cells, we show that the thrombin induced signaling cascade is independent of EGF receptor transactivation and rather relies on the activation of PKC to connect the increase of the intracellular Ca^2+^-concentration with the ERK signaling cascade.

## Conclusion

**Figure 12:**
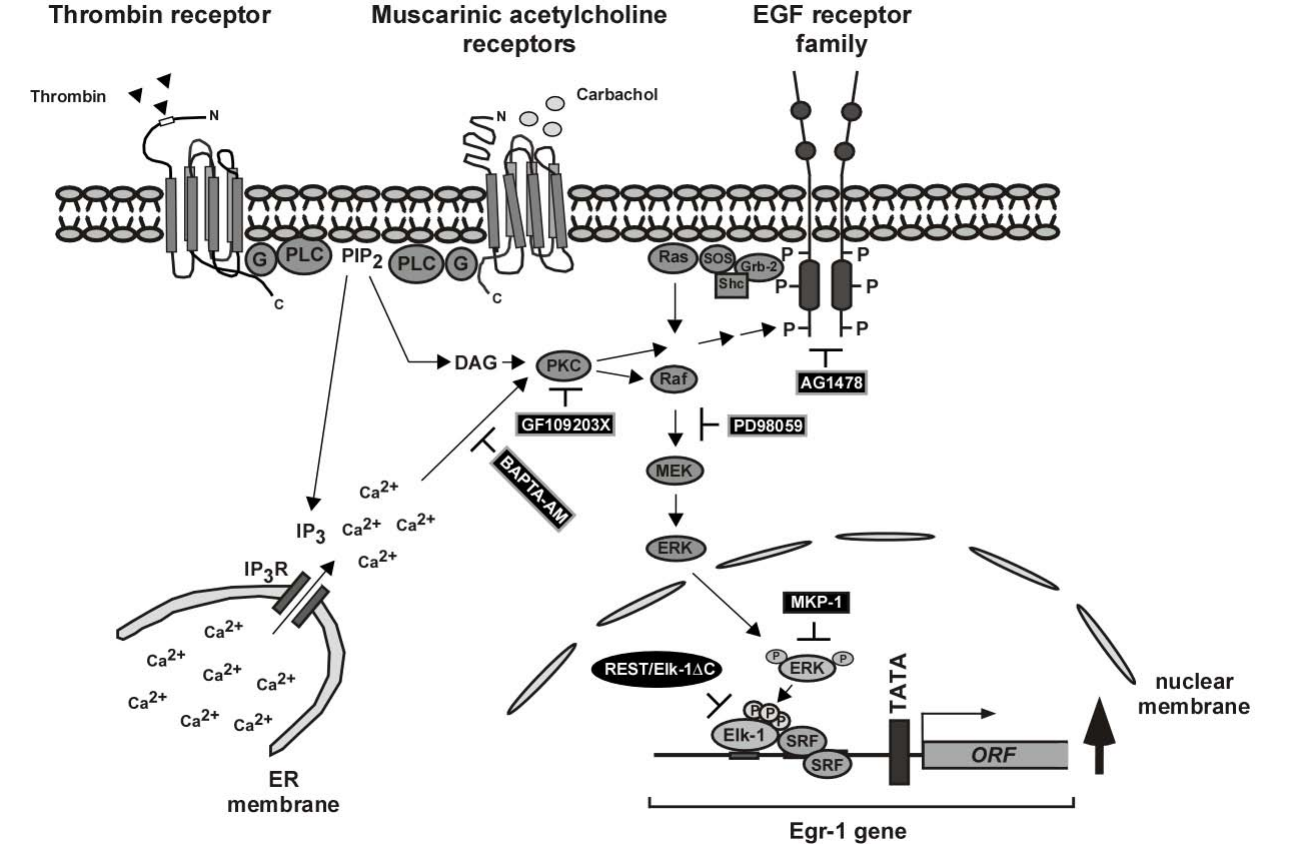
**Signaling pathway leading to Egr-1 biosynthesis in thrombin or carbachol-stimulated 39M1-81 cells**. Stimulation of protease-activated and M_1 _muscarinic acetylcholine receptors leads to the activation of phospholipase C (PLC), the generation of IP_3 _and the release of Ca^2+^-ions into the cytosol via stimulation of ionotropic IP_3 _receptors of the endoplasmic reticulum. The increase of the intracellular Ca^2+^-concentration is prevented by pretreating the cells with BAPTA-AM. Elevation of the intracellular Ca^2+^-concentration may induce a transactivation of the EGF receptor or an activation of PKC. Protein kinase C regulates directly or indirectly Raf activity. While preincubation of the cells with AG1478 was used to inhibit the tyrosine kinase activity of the EGF receptor, PKC activity was either blocked by incubation with the bisindolylmaleimide GF109203X or by prolonged treatment with TPA. Both transactivation of the EGF receptor or activation of PKC stimulated the ERK signaling pathway. The compound PD98059 was used to inhibit the phosphorylation of MAP kinase kinase by Raf, thus blocking the stimulus-induced phosphorylation and activation of ERK. Major nuclear substrates for ERK are ternary complex factors such as Elk-1, essential components of the serum response element ternary complex. The Egr-1 promoter contains five SREs that mediate signal-induced activation of Egr-1 gene transcription. Stimulus-induced Egr-1 biosynthesis was blocked by inhibiting ternary complex factor activity via expressing of a dominant-negative mutant (REST/Elk-1ΔC). Likewise, forced dephosphorylation of ERK by overexpression of MKP-1 blocked the signaling cascade leading to enhanced Egr-1 biosynthesis.

We have shown that stimulation of protease-activated receptors in 39M1-81 cells promotes the biosynthesis of Egr-1 via a signaling cascade involving elevated levels of intracellular Ca^2+^-ions, the activation of PKC and ERK, and the phosphorylation and activation of Elk-1. In the nucleus, Elk-1 connects the activation of ERK with the Egr-1 gene via SREs within the Egr-1 5'-upstream region. Overexpression of MKP-1 prevents the phosphorylation of Elk-1 and the subsequent transcription of the Egr-1 gene. The signaling cascade induced by thrombin or carbachol in 39M1-81 cells is summarized in the scheme depicted in Fig. 12. This study also shows that the dominant-negative Elk-1 mutant REST/Elk-1ΔC is a valuable tool to study ternary complex factor-regulated gene transcription.

## Methods

### Cell culture and transient transfections

39M1-81 cells, a kind gift of Jacques Pouysségur, Université de Nice, France, are derived from CCL39 cells, an established cell line of Chinese hamster lung fibroblasts, that express protease-activated receptors. CCL39 cells have been engineered to additionally express human type M_1 _muscarinic acetylcholine receptors [9]. The cells were cultured in the presence of 500 μg/ml G418. 39M1-81 cells expressing ΔRaf-1:ER were generated via retroviral gene transfer as previously described [42]. As a control, 39M1-81 cells were infected with a recombinant retrovirus encoding puromycin acetyltransferase, generating 39M1-81pac cells. 39M1-81ΔRaf-1:ER and 39M1-81pac cells were cultured in the presence of 1.5 μg/ml puromycin. The human brain glioma cell line 1321N1 was obtained from the European Collection of Cell Cultures (ECACC # 86030402) and cultured as described elsewhere [43]. SH-SY5Y cells were purchased from ATCC (# CRL-2266™) and maintained in Dulbecco's modified Eagles medium supplemented with 10% heat inactivated fetal calf serum, penicillin (100 U/ml) and streptomycin (100 μg/ml) at 37°C in 5% CO_2_. Cells were incubated for twenty-four hours in medium containing 0.05% serum before stimulation. Stimulation with thrombin (Calbiochem, Darmstadt, # 605195, dissolved in H_2_0 and used at a concentration of 1 U/ml), carbachol (Sigma # C-4382, dissolved in water and used at a concentration of 100 μM), and EGF (10 ng/ml, Promega, Mannheim, Germany, # G5021, dissolved in H_2_O as a 100 μg/ml stock solution) was performed as indicated. The MAP kinase kinase inhibitor PD98059 was purchased from Calbiochem (Darmstadt, Germany, # S513000), dissolved in DMSO and used at a concentration of 50 μM. Cells were preincubated with PD98059 for 6 hours. The EGF receptor-specific tyrosine kinase inhibitor AG1478 was also purchased from Calbiochem (# 658552), dissolved in DMSO and used at a final concentration of 0.5 μM. Cells were preincubated with AG1478 for 1 hour before stimulation. The PKC inhibitor bisindolylmaleimide (GF-109203X) was from Axxora (Lörrach, Germany, # 270-019-M005), dissolved in DMSO and used at a concentration of 10 μM as described [44]. Cells were preincubated with GF-109203X for 30 min. Stimulation with 12-*O*-tetradecanoylphorbol-13 acetate (TPA) (Calbiochem, Darmstadt, # 524400, dissolved in DMSO used at a final concentration of 100 ng/ml) was performed for twenty-four hours.

### Lentiviral gene transfer and reporter gene analysis

The lentiviral transfer vectors pFUW-MKP-1 and pFUW-REST/Elk-1ΔC have been described elsewhere [24,29,45]. The lentiviral transfer vectors pFWEgr-1.1luc and pFWEgr-1.2luc, encoding the luciferase reporter gene under the control of 239 or 490 nucleotides of the human Egr-1 5'-flanking region, and a lentiviral transfer vector encoding luciferase (pFUWluc2) under the control of the human ubiquitin C promoter have been described recently [22]. The transfer vector pFWEgr-1SREluc encodes the luciferase reporter gene under the control of the proximal SREs # 1 and 2 of the Egr-1 promoter. A minimal promoter was inserted downstream of the SREs consisting of a TATA box derived from the HIV long terminal repeat and an initiator element from the adenovirus major late promoter. The regulatory region of this transcription unit has been derived from plasmid pEgr-1SREluc [37]. To generate plasmid pFWEBS2^4^luc, a lentiviral transfer vector containing an Egr-1-responsive reporter gene, we exchanged the ubiquitin C promoter with the minimal promoter of plasmid pEBS2^4^luc [46]. The lentiviral transfer vector pFW9E3luc, encoding a luciferase reporter gene controlled by the chicken 9E3/cCAF promoter (sequence from -789 to +31, accession # NW001471685.1, nucleotides 13869435–13870254), was generated by inserting a HindIII fragment, derived from plasmid pGL3-9E3, upstream of the luciferase open reading frame. Plasmid pGL3-9E3 was a kind gift of Manuela Martins-Green, University of California, Riverside. The viral particles were produced as previously described [24] by triple transfection of 293T/17 cells with the *gag-pol-rev *packaging plasmid, the *env *plasmid encoding VSV glycoprotein and the transfer vector. Luciferase activities of infected cells were measured as described elsewhere [19]. Luciferase activities were normalized to the protein concentrations. Each experiment was repeated at least three times with consistent results.

### Preparation of cell extracts

Nuclear extracts and whole cell extracts were prepared as previously described [4].

### Western Blots

Nuclear extracts were prepared as described [4]. 20 μg of nuclear proteins were separated by SDS-PAGE and the blots were incubated with antibodies directed against Egr-1 (Santa Cruz, Heidelberg, Germany, #sc-110), Sp1 (Santa Cruz, Heidelberg, Germany, #sc-59), MKP-1 (Santa Cruz, Heidelberg, Germany, # sc-1199), or phosphorylated Elk-1 (Santa Cruz, Heidelberg, Germany, #sc-8406). To detect ERK and Phospho-ERK, 20 μg of proteins derived from whole cell extract preparations were separated on SDS-PAGE and transferred to nitrocellulose membranes. Blots were probed with an antibody directed against ERK (Santa Cruz, Heidelberg, Germany, #sc-153) or the phosphorylated forms of ERK1 and ERK2 (Cell Signaling, Frankfurt/Main, Germany, #9106). The ΔRaf:ER fusion protein was detected using an antibody directed against the murine estrogen receptor (Santa Cruz, Heidelberg, Germany, #sc-542).

## Abbreviations

Egr: early growth response; ERK: extracellular signal-regulated protein kinase; MKP: MAP kinase phosphatase; PKC: protein kinase C; SRE: serum response element.

## Authors' contributions

GT designed the study, generated the reporter plasmids, the lentiviral transfer vectors, and the 39M1-81-ΔRaf-1:ER cell line and drafted the manuscript. OGR performed all the experiments. Both authors read and approved the manuscript.
